# Preconditioning of bone marrow mesenchymal stem cells by prolyl hydroxylase inhibition enhances cell survival and angiogenesis in vitro and after transplantation into the ischemic heart of rats

**DOI:** 10.1186/scrt499

**Published:** 2014-09-25

**Authors:** Xian-Bao Liu, Jian-An Wang, Xiao-Ya Ji, Shan Ping Yu, Ling Wei

**Affiliations:** Department of Cardiology, Second Affiliated Hospital, Zhejiang University School of Medicine, Hangzhou, 310016 China; Department of Anesthesiology, Emory University School of Medicine, 101 Woodruff Circle, Woodruff Memorial Research Building Suite 617, Atlanta, GA 30322 USA

## Abstract

**Introduction:**

Poor cell survival and limited functional benefits have restricted the efficacy of bone marrow mesenchymal stem cells (BMSCs) in the treatment of myocardial infarction. We showed recently that hypoxia preconditioning of BMSCs and neural progenitor cells before transplantation can enhance the survival and therapeutic properties of these cells in the ischemic brain and heart. The present investigation explores a novel strategy of preconditioning BMSCs using the Hypoxia-inducible factor 1α (HIF-α) prolyl hydroxylase inhibitor dimethyloxalylglycine (DMOG) to enhance their survival and therapeutic efficacy after transplantation into infarcted myocardium.

**Methods:**

BMSCs from green fluorescent protein transgenic rats were cultured with or without 1 mM DMOG for 24 hours in complete culture medium before transplantation. Survival and angiogenic factors were evaluated *in vitro* by trypan blue staining, Western blotting, and tube formation test. In an ischemic heart model of rats, BMSCs with and without DMOG preconditioning were intramyocardially transplanted into the peri-infarct region 30 minutes after permanent myocardial ischemia. Cell death was measured 24 hours after engraftment. Heart function, angiogenesis and infarct size were measured 4 weeks later.

**Results:**

In DMOG preconditioned BMSCs (DMOG-BMSCs), the expression of survival and angiogenic factors including HIF-1α, vascular endothelial growth factor, glucose transporter 1 and phospho-Akt were significantly increased. In comparison with control cells, DMOG-BMSCs showed higher viability and enhanced angiogenesis in both *in vitro* and *in vivo* assays. Transplantation of DMOG-BMSCs reduced heart infarct size and promoted functional benefits of the cell therapy.

**Conclusions:**

We suggest that DMOG preconditioning enhances the survival capability of BMSCs and paracrine effects with increased differentiation potential. Prolyl hydroxylase inhibition is an effective and feasible strategy to enhance therapeutic efficacy and efficiency of BMSC transplantation therapy after heart ischemia.

## Introduction

Myocardial infarction (MI) is a leading cause of congestive heart failure that results from irreversible loss of cardiomyocytes, scar formation and ventricular remodeling [1]. A large amount of animal and clinical research has demonstrated that bone marrow mesenchymal stem cells (BMSCs) are capable of improving the post-MI recovery due to continued self-renewal, transdifferentiation and paracrine effects [2]. However, the morphological and functional benefits of BMSC therapy are limited primarily due to the low survival rate of transplanted cells in the ischemic host environment [3]. Strategies to improve BMSC tolerance to hypoxic/ischemic conditions as well as their functional benefits are therefore critically needed for clinical translation of the cell therapy.

Earlier work demonstrated that pretreatment of adult BMSCs with cardiomyogenic growth factors before transplantation improves the cells’ *in vivo* cardiac differentiation as well as functional recovery in a dog model of the chronically infarcted myocardium [4]. In a recent study, a recombinant cocktail consisting of transforming growth factor beta-1, bone morphogenetic protein-4, activin A, retinoic acid, insulin-like growth factor-1, fibroblast growth factor-2, α-thrombin, and interleukin-6 was formulated to engage human mesenchymal stem cells into cardiopoiesis. Compared with unguided counterparts, cardiopoietic mesenchymal stem cells delivered into infarcted myocardium achieved superior functional and structural benefits [5]. In order to enhance cell survival and repair potential, some studies have overexpressed anti-apoptotic and trophic/growth factors in stem cells or progenitor cells [6–9]. This molecular biological approach can effectively reduce cell death; however, it causes a practical concern for increased risk of tumor growth due to lasting and high expression of anti-apoptotic factors. Alternatively, we recently explored a new approach of hypoxic preconditioning by exposing stem cells and progenitor cells to sublethal hypoxia before transplantation [10]. This hypoxic preconditioning increased the expression of prosurvival and proangiogenic factors including hypoxia-inducible factor (HIF)-1α, angiopoietin-1, vascular endothelial growth factor (VEGF) and its receptor Flk-1, erythropoietin (EPO), Bcl-2, and Bcl-xL. Cell death and caspase-3 activation in these cells was significantly lower compared with that in normoxic cells both *in vitro* and after transplantation. Transplantation of hypoxic preconditioned BMSCs, neural progenitors or cardiac progenitors, resulted in greater cell survival, angiogenesis, better tissue repair and enhanced functional recovery after myocardial and cerebral ischemia [7, 10–18].

It is suggested that a preconditioning procedure sets cells into a primed state before they encounter the harsh microenvironment of hypoxia/ischemia and elevated levels of injurious factors [19, 20]. At present, the preconditioning strategy has been increasingly accepted and applied in cell-based transplantation therapy and shows multiple therapeutic benefits after ischemic disorders in the central nervous system and peripheral organs [21–23]. Besides hypoxic preconditioning, we and others have shown that stem cells/neural progenitors can be preconditioned by some endogenous factors and neural peptides such as growth factors, interleukin-6, apelin and EPO [4, 17, 18, 24–26]. Similarly, pharmacological preconditioning has emerged as a means of priming transplanted cells. For example, pretreatment of skeletal myoblasts and mesenchymal stem cells with diazoxide achieved protective and functional benefits in the treatment of cardiomyocyte ischemia [27, 28]. Diazoxide pretreatment also protects neurons from glutamate toxicity [29].

Prolyl hydroxylases are members of an iron-dependent and 2-oxoglutamate-dependent dioxygenase enzyme family. The HIF prolyl hydroxylase enzymes, termed the prolyl hydroxylase domain, play an important role in oxygen regulation in different tissues and organs [30]. Cells recognize and respond to hypoxia by accumulating the transcription factor HIF-1, composed of oxygen-sensitive inducible HIF-1α and constitutive HIF-1β subunits. Prolyl hydroxylase domain enzymes are involved in the degradation of the HIF-1α subunit. Under hypoxic conditions, the lack of oxygen leads to stabilization of HIF-1α to form a HIF heterodimer that is subsequently translocated to the nucleus, triggering the transactivation of target genes. The nature of the target gene and type of expressed proteins may vary depending upon the type of tissues and disease conditions [31]. Similar effects can be obtained using the prolyl hydroxylase inhibitor, dimethyloxalylglycine (DMOG). HIF-1α in turns activates the transcription of a number of angiogenic and survival genes such as VEGF, glucose transporter 1 (Glut-1), and EPO [32–36]. Our previous study showed that DMOG enhanced cell survival against the apoptotic insult of serum deprivation in a dose-dependent manner in BMSC cultures, mediated by the mechanisms of HIF-1α stabilization, regulation of the mitochondrial pathway and phosphatidylinositol-4,5-bisphosphate 3-kinase (PI3K)/Akt activation [37]. DMOG treatment reduced mitochondrial cytochrome c release, prevented nuclear translocation of apoptosis inducing factor, and promoted Akt phosphorylation [37]. In a subsequent investigation, we demonstrated in neuronal cultures that DMOG (100 μM) reduced cell death induced by oxygen glucose deprivation [34]. In a cerebral ischemic stroke model, DMOG treatment (50 mg/kg, intraperitoneally) enhanced HIF-1α activation and transcription of its downstream genes VEGF, EPO, endothelial nitric oxide synthesis, and pyruvate dehydrogenase kinase-1. The DMOG treatment reduced caspase-3 activation and infarct formation and improved functional recovery after stroke [34]. These data suggest that the oxygen-sensing pathway is a potent protective mechanism. The present investigation explores the hypothesis that DMOG, as an inhibitor of the oxygen-regulated enzyme prolyl hydroxylase, can mimic the hypoxic environment to prime BMSCs for an effective transplantation therapy after heart ischemia.

## Methods and materials

### Bone marrow mesenchymal stem cell cultures

BMSCs from green fluorescent protein transgenic rats were isolated and harvested as described previously [38]. In brief, bone marrow tissues were acquired by flushing the cavities of femurs and tibias with basal Dulbecco’s modified Eagle’s medium. Collected bone marrow cells were seeded into flasks with Dulbecco’s modified Eagle’s medium supplemented with 10% fetal bovine serum. Cells were cultured at 37°C in a humidified environment with 5% carbon dioxide. Nonadherent cells were removed 24 hours later, and adherent cell colonies were washed three times with phosphate-buffered saline solution (PBS). Fresh complete medium was added and changed every 3 to 4 days. Cells were subcultured 1:2 or 1:3 when they reached 80% confluence. BMSCs of four to six passages were used in this study.

For identification of BMSCs, cell surface markers CD90, CD34 and CD45 were detected by fluorescence-activated cell sorting. The detailed characterization of our isolated bone marrow cells and multipotency of identified BMSCs were confirmed in our previous investigations [39]. In the present investigation, CD90^+^/CD34^–^/CD45^–^ cells were selected and tested.

### Dimethyloxalylglycine preconditioning of BMSCs

Cells were subcultured at 1:2/1:3 and cultured for 2 to 3 days until they reached 70 to 80% confluence. Cells were then exposed to fresh complete medium supplemented with 1 mM DMOG for 24 hours. The DMOG concentration was selected based on our previous investigations [37]. For the nonpreconditioned control, culture medium was changed to fresh complete medium at the same time as DMOG treatment. Before transplantation, cells were labeled with Hoechst 33342 by adding a final concentration of 10 μM/ml in the culture medium and were incubated for 2 hours to trace BMSCs after transplantation. BMSCs were then washed six times with PBS to remove unbound Hoechst dye, and digested with 0.25% (w/v) trypsin ethylenediamine tetraacetic acid, followed by suspension in complete medium. After several centrifugations and PBS washes, cells were suspended in a serum-free medium at 1 × 10^6^ cells per 150 μl. This solution was injected into five sites (30 μl each) in peri-infarct myocardium 30 minutes after the ligation of the left anterior descending coronary artery as described previously [40]. For control, MI rats received the same volume of serum-free/cell-free medium. Four groups of 10 rats each were randomly divided as follows: sham-operated control group; MI with injection of medium as MI-medium control; MI with transplantation of nonpreconditioned BMSCs as N-BMSC control; and MI with transplantation of DMOG-pretreated BMSCs as DMOG-BMSC control.

### Myocardial infarction model of rats

All animal experiments and surgical procedures were approved by the University Animal Research and Use Committee (IACUC, Emory University; No. 2001421) and met National Institutes of Health standards. Wistar rats were subjected to general anesthesia with 4% chloral hydrate (4 mg/kg intraperitoneally) and ventilated with room air using a small animal ventilator (Vetronics, Lafayette, IN, USA). MI was induced by ligation of the left anterior descending coronary artery with a 6–0 silk suture [10, 40]. Successful performance of coronary artery occlusion was verified by the blanching of the myocardium distal to the coronary ligation.

### Cell death assessments of Trypan Blue staining *in vitro*

To evaluate the protective effect of the DMOG preconditioning *in vitro*, cell death induced by hydrogen peroxide (100 μM) in a serum-free medium (90 minutes) was tested. This combination insult was intended to mimic the microenvironment of a myocardial ischemic attack [41]. Trypan Blue staining was applied to assess cell death*.* Serum-free medium containing 0.05% Trypan Blue was added for 10 minutes and phase contrast images were taken in five randomly chosen fields per well. The cell death percentage was determined by the ratio of Trypan Blue-positive cells to the total number of cells.

### Terminal deoxynucleotidyl transferase biotin-dUPT nick end labeling in heart sections

The terminal deoxynucleotidyl transferase biotin-dUPT nick end labeling (TUNEL) staining kit (DeadEnd™ Fluorometric TUNEL system; Promega, Madison, WI, USA) was used for detecting cell death in heart sections. After fixing with 10% buffered formalin phosphate (Fisher Scientific, Pittsburgh, PA, USA) for 10 minutes, heart sections were pretreated with -20°C ethanol:acetic acid (2:1) and 0.2% Triton X-100. The slices were then incubated with an equilibration buffer as specified by the TUNEL kit. Sequentially, the TdT enzyme and nucleotide mix were added at proportions instructed by the kit for 75 minutes in a humidified chamber at room temperature. The slices were then washed with 2× SSC washing buffer for 15 minutes, and finally washed three times with PBS. In the end, the sections were slip covered and examined under a florescent microscope.

### Western blot analysis

Cells were harvested and lysed with modified RIPA buffer (50 mM HEPES, pH 7.3, 1% sodium deoxycholate, 1% Triton X-100, 0.1% SDS, 150 mM NaCl, 1 mM ethylenediamine tetraacetic acid, 1 mM Na_3_VO_4_, 1 mM NaF, and protease inhibitor cocktail (Roche, Nutley, NJ, USA)). Cells were vortexed repeatedly until completely lysed, followed by centrifugation at 14,000 × *g* for 20 minutes. The concentration of proteins from different groups was determined using the Bicinchoninic Acid Assay (Sigma, St Louis, MO, USA). All of the above steps were performed at 4°C.

Proteins (30 to 50 μg) per sample were electrophoresed on a 6 to 18% gradient gel by SDS-PAGE in a Hoefer Mini-Gel system (Amersham Biosciences, Piscataway, NJ, USA) and transferred in a Hoefer Transfer Tank (Amersham Biosciences) to a PVDF membrane (BioRad, Hercules, CA, USA). After blockage by 5% milk in Tris-buffered saline with 0.1% Tween at room temperature for 2 hours, membranes were incubated with specific primary antibodies overnight at 4°C. Alkaline phosphatase/ horseradish peroxidase-marked secondary antibodies were then conjugated at room temperature for 2 hours. Finally, the expression signals were detected with BCIP/NBT solution (Sigma) and analyzed by Image-Pro Plus software (NIH, Bethesda, MD, USA).

### Tube formation test

The tube formation test is a well-established assay used to detect the formation of three-dimensional vessels and used widely to assess angiogenesis *in vitro.* Matrigel (Sigma) was prepared in 24-well plate according to the manufacturer's instructions. DMOG-preconditioned (DMOG-BMSCs) or nonpreconditioned BMSCs were seeded into the coated wells at a density of 50,000 cells, and were incubated for 6 hours at 37°C for full development of vessel-like tube structures. Tube formation was quantified by the cumulative tube length. In the case where several tube-like structures merged together or branched, the total length of tubes was calculated as the sum of the length of the individual branches.

### Immunofluorescence staining

For immunofluorescence staining, heart slices were fixed with 10% formalin for 10 minutes, followed by permeabilization with 0.2% Triton X-100 for 5 minutes and blocked with 1% fish gelatin (Sigma) for 1 hour at room temperature. Specific primary antibodies were incubated overnight at 4°C in a humidified environment, and washed three times with PBS. Specimens were then incubated with Cy3-conjugated Donkey anti-rabbit IgG (1:500; Jackson ImmunoResearsh, West Grove, PA, USA) or Alexa Fluor 488 anti-goat IgG (1:200; Molecular Probes, Carlsbad, CA, USA) for 1 hour at room temperature. Nuclear staining was performed by treatment with Hoechst 33342 (1:20,000; Molecular Probes, Carlsbad, CA, USA) for 5 minutes. Slices were then mounted and observed under a florescent microscope (BX51: Olympus, Center Valley, PA, USA).

### Evaluation of cardiac function

Transthoracic echocardiography and hemodynamic measurement were performed for the analysis of cardiac function 4 weeks after myocardial ischemia. Rats were anesthetized with 4% chloral hydrate by intraperitoneal injection. Echocardiography was performed using a VisualSonics Vevo 2100 system (VisualSonics, Inc., Toronto, ON, Canada) with a 12.0 MHz transducer. The left ventricular end-diastolic diameters and left ventricular end-systolic diameters were measured by two-dimensional targeted M-mode, and the left ventricular ejection fraction (LVEF) was calculated:


Using echocardiographic evaluation, hemodynamic measurement was performed. After right carotid artery exposure, a microtip catheter was cannulated into the left ventricle and was connected with a MLT0699 disposable pressure transducer (AD Instrument, Colorado Springs, CO, USA). The left ventricular systolic pressure (LVSP), left ventricular end-diastolic pressure (LVEDP), maximum change rate of left ventricular pressure rise and fall (±dp/dt), time constant of the isovolumic pressure decline (Tau) and heart rate were monitored and recorded using the Powerlab/800 data acquisition system (AD Instrument).

### Measurement of infarct size

Rats were sacrificed after measuring hemodynamics by an overdose of anesthetic. Hearts were harvested quickly and split into transverse sections: apex, mid left ventricle, and base. The tree sections of the hearts were embedded in optimal cutting temperature compound (Sakura Finetek USA Inc., Torrance, CA, USA). Samples cut at 10 μm thickness were stained with Masson’s trichrome and images of each slide were digitized through the NIH image analysis system. The percentage of infarct size/fibrotic area was calculated by dividing the sum of epicardial and endocardial circumference of the infarcted area by the sum of the total endocardial and epicardial circumference of the left ventricle [42].

### Statistical analysis

Data were analyzed with SPSS 13.0 (IBM Corp. Armonk, NY, USA) and expressed as mean ± standard error of mean. Student’s two-tailed *t* test was applied for comparison of two independent experimental groups and one-way analysis of variance followed by the Tukey *post hoc* test for multiple comparisons. Statistical significance was defined as *P* <0.05.

## Results

### Upregulation of survival and angiogenic factors in DMOG-preconditioned BMSCs

**Figure 1:**
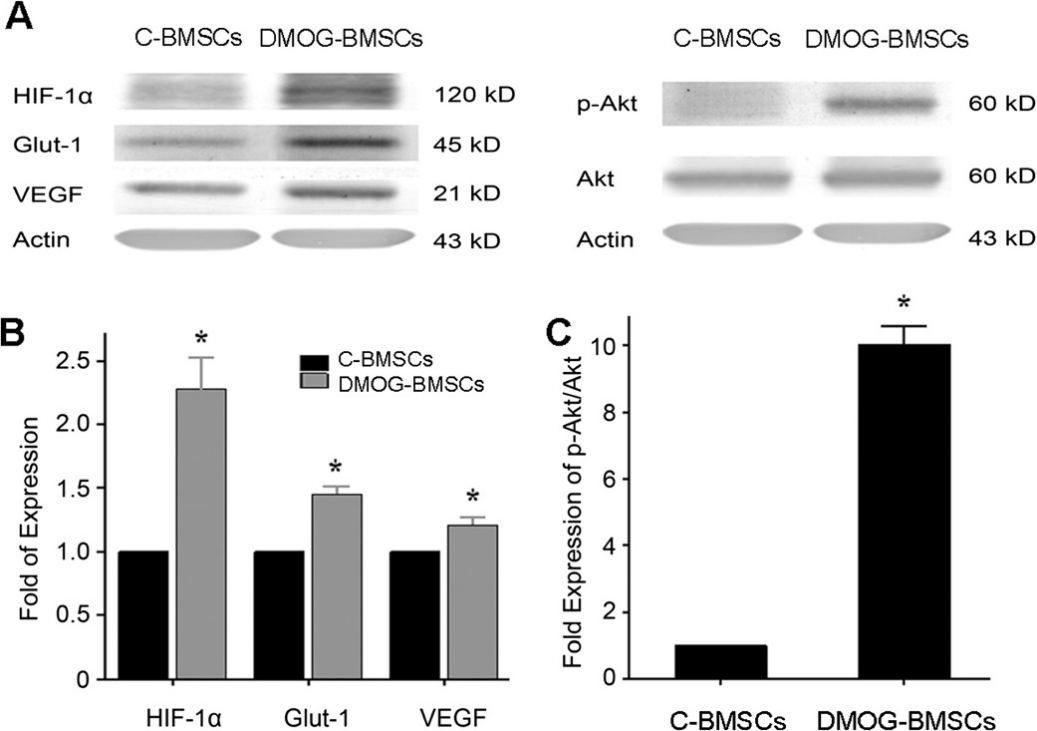
**Effect of dedimethyloxalylglycine preconditioning on the expression of prosurvival and angiogenic factors. (A)** Rat bone marrow mesenchymal stem cells (BMSCs) were cultured with or without dedimethyloxalylglycine (DMOG, 1 mM) for 24 hours. Hypoxia-inducible factor (HIF)-1ɑ, glucose transporter 1 (Glut-1), vascular endothelial growth factor (VEGF) and phospho-Akt were then detected by western blotting. Beta-actin was used as the loading control. **(B)** Densitometric analysis was applied for comparison of the relative expression levels of different proteins in DMOG-BMSCs with respect to vehicle control BMSCs (C-BMSCs) that is arbitrarily presented as 1. *n* =3 independent assays. *P <0.05 compared with C-BMSC group. **(C)** Ratio of phospho-Akt against total Akt expression, quantified from the western blot gels. A large increase of 10-fold Akt phosphorylation was seen in DMOG-BMSCs compared with control BMSCs. *n* =3 independent assays. *P <0.05 vs. control cells.

To evaluate the effect of DMOG preconditioning on BMSCs, we first analyzed the expression of survival and angiogenic proteins in control BMSCs (C-BMSCs) and DMOG-treated BMSCs (DMOG-BMSCs) using western blot analysis. HIF-1α, VEGF, Glut-1 and phospho-Akt were detected in both groups of BMSCs. However, significantly higher expressions of HIF-1α and the downstream factors Glut-1 and VEGF were seen in DMOG-BMSCs compared with C-BMSCs. Akt activation was markedly increased by DMOG preconditioning so that the ratio of phospho-Akt/Akt increased approximately 10-fold compared with C-BMSCs (Figure 1).

### Dimethyloxalylglycine preconditioning reduced BMSC cell death *in vitro* and after transplantation

**Figure 2:**
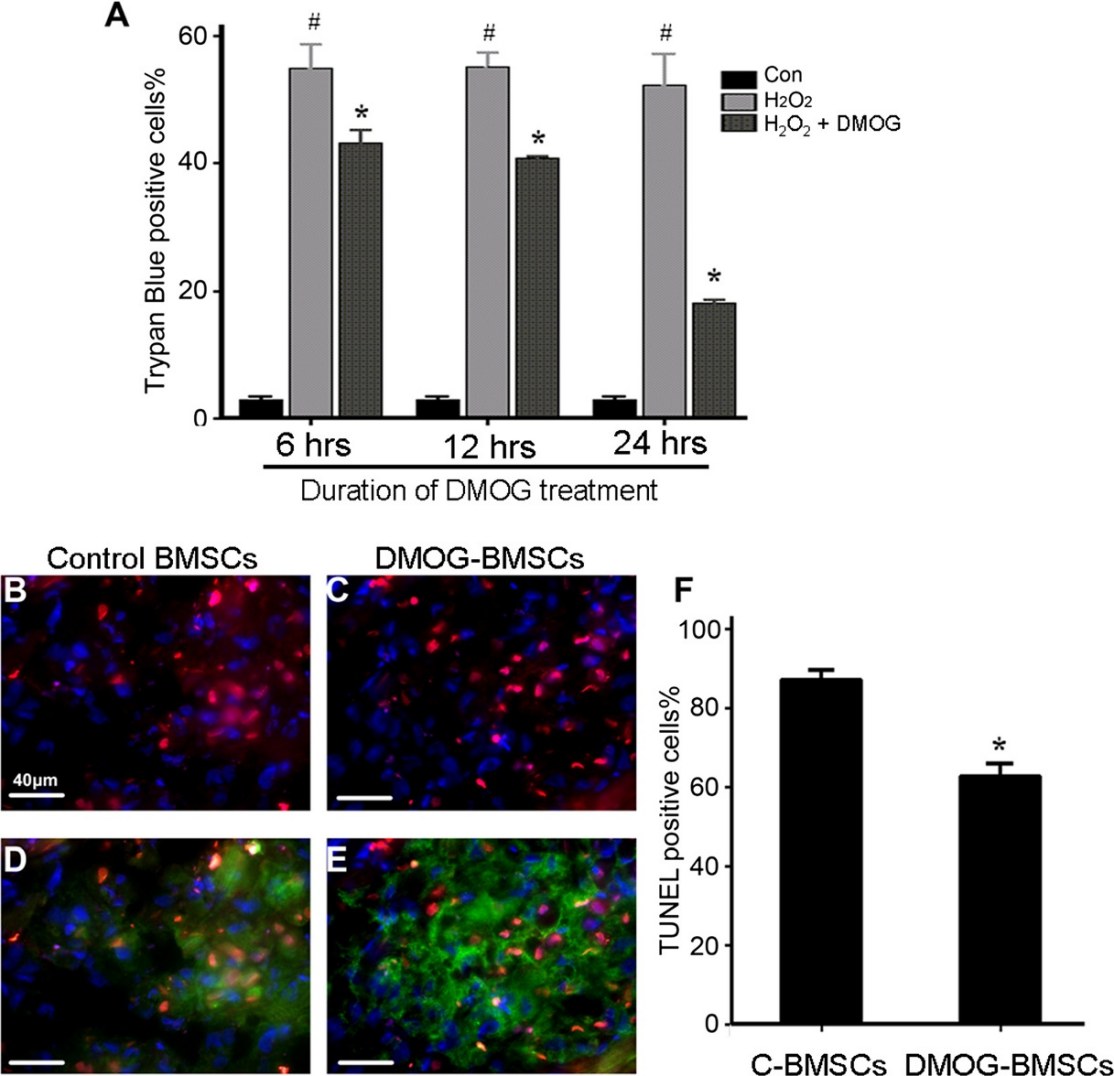
**Effect of dedimethyloxalylglycine preconditioning on cell death** ***in vitro*** **and after transplantation. (A)** Hydrogen peroxide (H_2_O_2_)-induced cell death was measured in bone marrow mesenchymal stem cell (BMSC) cultures with and without dedimethyloxalylglycine (DMOG) preconditioning. After different durations of DMOG (1 mM) treatment, cell death was induced by H_2_O_2_ (100 μM) in a serum-free medium for 90 minutes. The membrane-permeable dye Trypan Blue was added 5 to 10 minutes before cell death measurement. Damaged BMSCs were identified as cells unable to exclude the blue color dye from the cytosol. *n* =3 independent tests in each time group. *P <0.05 compared with C-BMSC group. ^#^ *P* <0.05 compared with basal control group. **(B)** to **(E)** Green fluorescent protein (GFP)-positive BMSCs (green) were labeled with Hoechst 33342 (blue) before transplantation to further facilitate tracking transplanted cells. Cell death was evaluated 24 hours after transplantation using terminal deoxynucleotidyl transferase biotin-dUPT nick end labeling (TUNEL) staining (red) in heart sections. Co-labeling of GFP/Hoechst/TUNEL fluorescence was designated as transplanted dead BMSCs. **(F)** Summarized cell count data of GFP/Hoechst/TUNEL-positive cells against total exogenous (GFP/Hoechst-positive) cells. *n* =4 independent experiments in each group. *P <0.05 compared with C-BMSC group.

To examine whether DMOG-induced preconditioning could increase the tolerance of BMSCs, cell death was first examined in BMSC cultures, which helped to determine the protective effect of different durations of DMOG pretreatment. In the Trypan Blue assay, hydrogen peroxide (6 to 24 hours) caused significantly more than 50% cell death in C-BMSC cultures, while DMOG pretreatment of 6, 12 and 24 hours showed time-dependent protective effects. Trypan Blue-positive cells were significantly reduced in DMOG-treated cells, with the strongest protection induced by 24-hour DMGO pretreatment (Figure 2). The cell death rate in this group decreased from 52.4 ± 4.8% to 18.1 ± 0.4% (*n* =4, *P* <0.05). Based on this observation, we selected the 24-hour DMOG exposure in our preconditioning procedure in the following experiments.

To evaluate whether DMOG-BMSCs could show enhanced survival *in vivo*, C-BMSCs and DMOG-BMSCs were implanted into the peri-infarct region 30 minutes after the left anterior descending ligation in rats. Animals were sacrificed 24 hours later to identify the fate of transplanted BMSCs. This time point was selected because the majority of cell death after transplantation occurred within 24 hours [3]. Cell death was identified by the ratio of TUNEL/Hoechst/green fluorescent protein co-labeled cells versus total Hoechst/green fluorescent protein-positive cells (Figure 2B,C,D,E,F,G). In the C-BMSC group, 87.3 ± 2.2% of total transplanted cells were TUNEL-positive. While transplanted DMOG-BMSCs showed significantly less death, there were 62.8 ± 3.1% TUNEL-positive cells among the total transplanted cells (Figure 2F).

### Dimethyloxalylglycine preconditioning enhanced angiogenic activities of BMSCs *in vitro* and after transplantation

**Figure 3:**
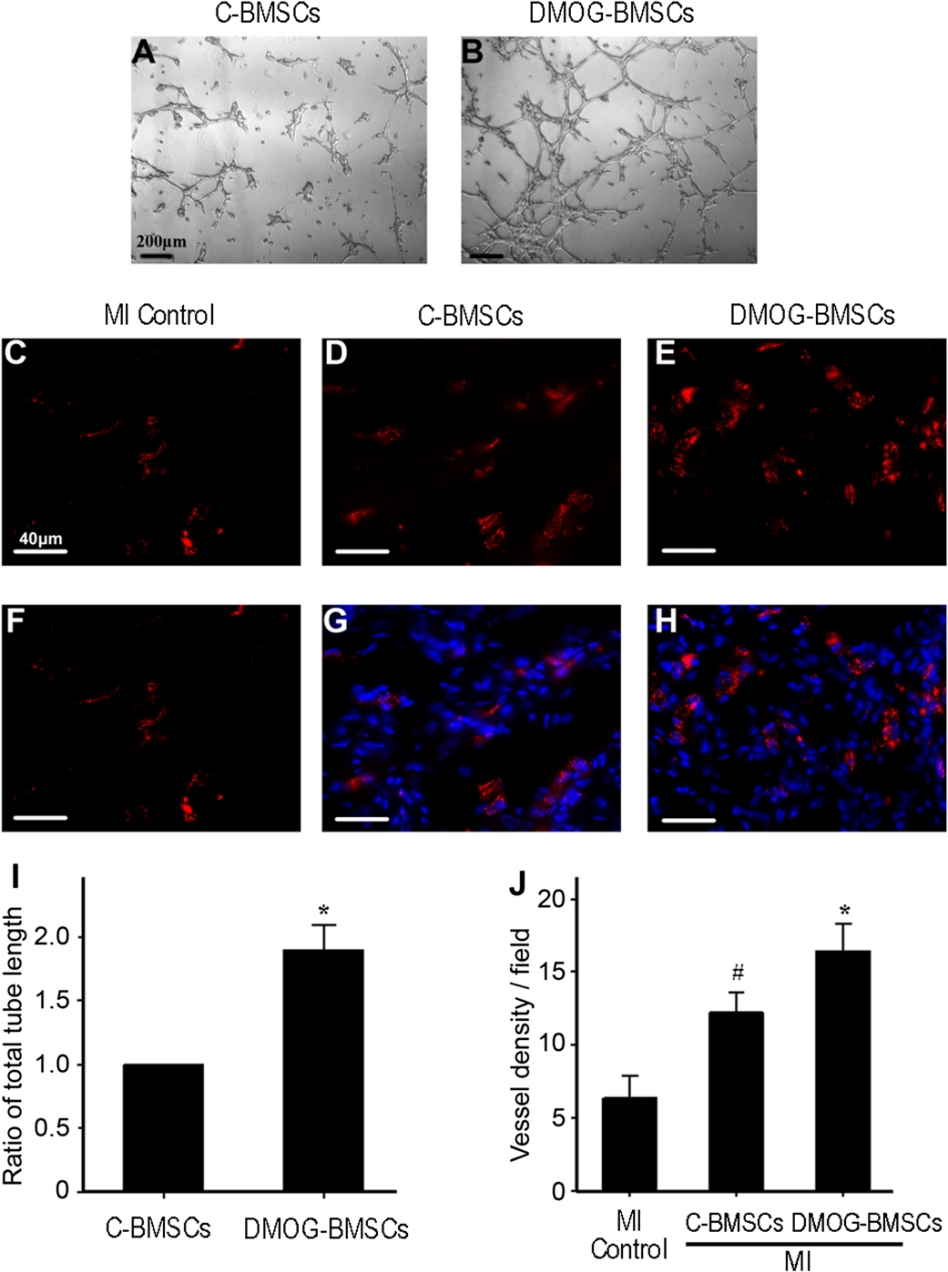
**Effect of dedimethyloxalylglycine preconditioning on angiogenesis** ***in vitro*** **and after transplantation. (A)**, **(B)** Tube formation test stimulated by Matrigel was performed to identify the angiogenic activity of control bone marrow mesenchymal stem cells (C-BMSCs) and dedimethyloxalylglycine-preconditioned BMSCs (DMOG-BMSCs) *in vitro*. **(C)** to **(H)** Angiogenesis was inspected using von Willebrand factor staining (red) in heart sections from the myocardial infarction (MI), C-BMSC and DMOG-BMSC groups 4 weeks after MI. Hoechst staining (blue) shows the total cells. **(I)** Summary of total tube length measured in **(A)** and **(B)**. The total tube length in the C-BMSC group was arbitrarily presented as 1. *n* =3 independent measurements. **(J)** Summary of total vessel density in different groups of *in vivo* experiments. *n* =8 animals in each group. *P <0.05 compared with C-BMSC group; ^#^ *P* <0.05 compared with MI control group.

The effect of DMOG preconditioning on BMSC-induced angiogenesis was examined *in vitro* and *in vivo*. For *in vitro* studies, tube formation stimulated by Matrigel was tested. Although C-BMSCs were able to form vessel tubes in response to Matrigel, DMOG-BMSCs formed more tubes and the total tube length was significantly longer (1.9-fold) than that formed by C-BMSC (Figure 3A,B,I). To assess angiogenic activity after transplantation, we stained the endothelial marker von Willebrand factor in heart sections. Both BMSC transplantation groups presented increased vessel density/area compared with the MI control group. Transplantation of DMOG-BMSCs showed even greater angiogenesis in the ischemic heart. More vessels were observed in the DMOG-BMSC group compared with the C-BMSC group (Figure 3C,D,E,F,G,H,J).

### Reduced infarct size after transplantation of BMSCs

**Figure 4:**
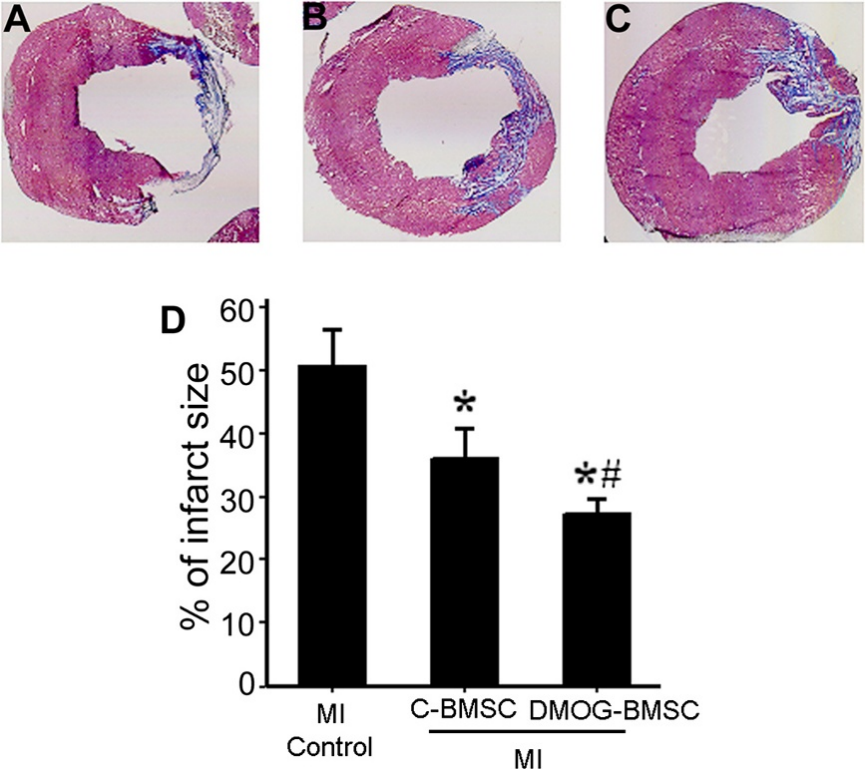
**Effect of bone marrow mesenchymal stem cell transplantation on ischemia-induced infarct formation.** Heart infarct area and scar formation were determined using Masson’s Trichrome staining 4 weeks after myocardial infarction (MI). **(A)** to **(C)** Images of representative infarcted hearts from a MI control rat, a MI rat receiving control bone marrow mesenchymal stem cells (C-BMSCs), and a MI rat receiving dedimethyloxalylglycine-preconditioned BMSCs (DMOG-BMSCs). **(D)** Transplantation of BMSCs reduced heart infarction formation; the protective effects were significantly greater with transplantation of DMOG-BMSCs. *n* =5 rats in each group. *P <0.05 compared with MI group; ^#^ *P* <0.05 compared with C-BMSC group.

Masson’s trichrome staining was used to assess fibrosis and scar formation 4 weeks after myocardial infarction and BMSC transplantation. Obvious infarct and scar formation was observed in the ischemic hearts that received cell-free medium injection. In comparison, the infarct size in both C-BMSC and DMOG-BMSC implantation groups was significantly smaller. Transplantation of DMOG-BMSCs resulted in the smallest infarct size in the ischemic heart (Figure 4).

### Improved cardiac function after BMSC transplantation

**Figure 5:**
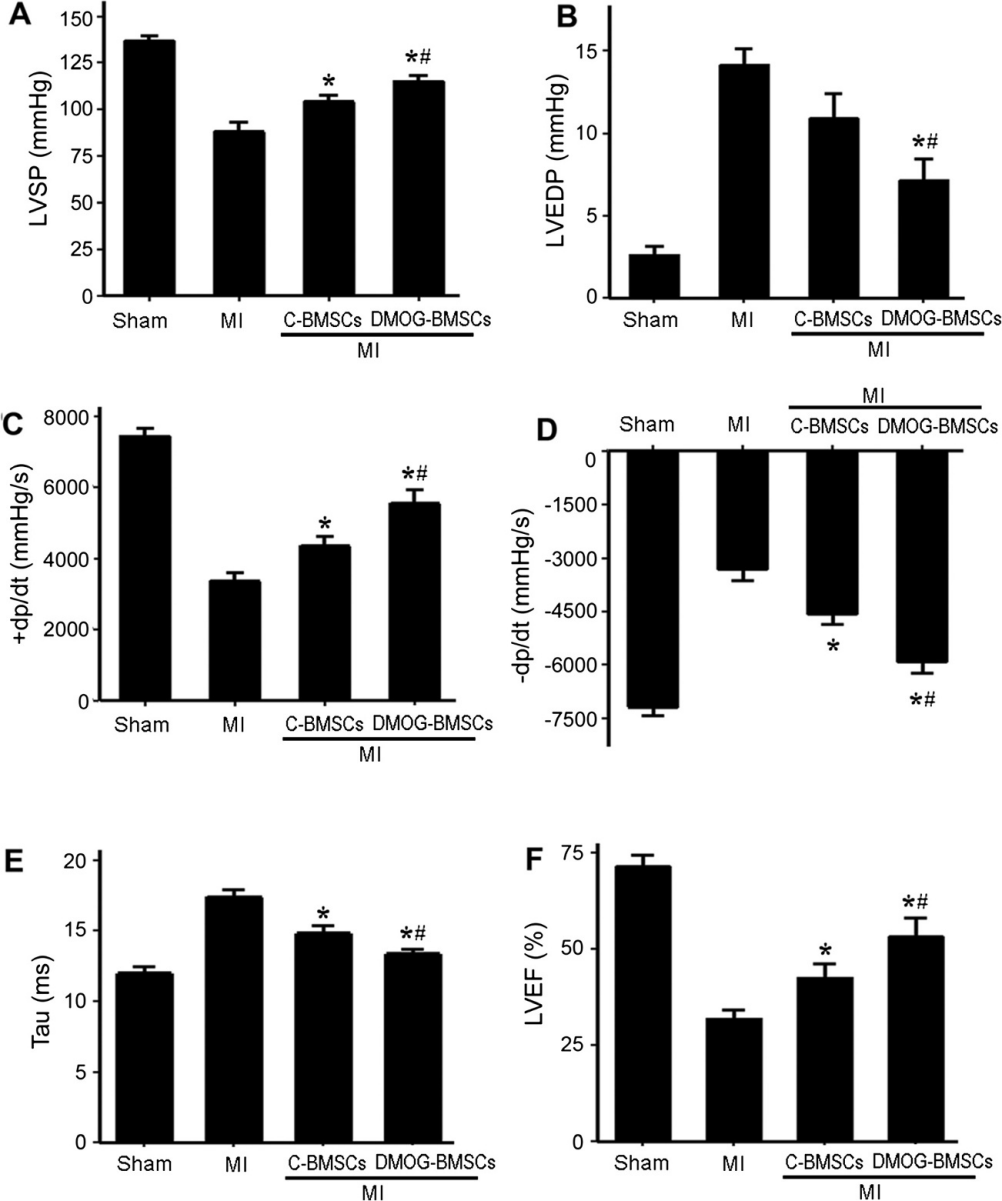
**Effect of bone marrow mesenchymal stem cell transplantation on functional recovery after heart ischemia.** Heart function was measured 4 weeks after myocardial infarction (MI) and cell transplantation treatment. MI rats showed functional deficits in all measurements of **(A)** left ventricular systolic pressure (LVSP), **(B)** left ventricular end-diastolic pressure (LVEDP), **(C)** maximum change rate of left ventricular pressure rise and fall (±dp/dt), **(D)** minimum change rate of left ventricular pressure rise and fall (–dp/dt), **(E)** time constant of the isovolumic pressure decline (Tau) and **(F)** left ventricular ejection fraction (LVEF). BMSC transplantation improved functional parameters of MI rats. Rats received dedimethyloxalylglycine-preconditioned BMSCs (DMOG-BMSCs) showed the enhanced functional recovery in all four functional parameters. *n* =8 rats in each group. *P <0.05 compared with MI-only group. ^#^ *P* <0.05 compared with the control BMSC (C-BMSC) group.

The ischemic heart functional recovery after BMSC transplantation was measured by the changes in LVSP, LVEDP, +dp/dt, -dp/dt, Tau and LVEF 4 weeks after ischemia and treatments. Compared with the MI control group, transplantation of both C-BMSCs and DMOG-BMSCs facilitated better cardiac recovery, shown as increased LVSP and absolute values of ± dp/dt, and decreased Tau and promotion of LVEF (Figure 5). However, C-BMSC transplantation had no effect on the value of LVEDP, while DMOG-BMSC transplantation significantly reduced the LVEDP value to a near-normal level. Furthermore, even better recovery shown as significantly higher LVSP, enhanced ± dp/dt, lower LVEDP and TAU, and ameliorated LVEF was seen in MI rats receiving DMOG-BMSCs (Figure 5).

## Discussion

The present investigation demonstrates for the first time that the prolyl hydroxylase inhibitor DMOG can be used to prime BMSCs in cell-based transplantation therapy after heart ischemia. We show that DMOG-pretreated BMSCs are more resistant to death both *in vitro* and after transplantation into the ischemic heart. DMOG-BMSCs show enhanced angiogenic activities. Survival and angiogenic factors such as HIF-1α, VEGF, Glut-1 and phospho-Akt increased after DMOG preconditioning, a typical gene regulation usually seen with hypoxic preconditioning. Transplantation of DMOG-BMSCs also leads to better recovery of the heart function in the rat’s MI model. It is thus likely that, as a regulator of the oxygen sensing system, DMOG can be used as an alternative way of preconditioning stem cells/progenitors. Like hypoxic preconditioning, DMOG preconditioning can optimize BMSC viability and regenerative capability for better engraftment and/or functional benefit in the harsh environment of myocardial infarction.

We and others have shown that the prolyl hydroxylase inhibitor DMOG prevents acute damage in the ischemic heart and brain as well as ameliorating BMSC cell death under pathological conditions [34, 37, 43]. However, DMOG has not been tested as a preconditioning reagent for stem cell therapy. So far, there has been only one related report showing that intraperitoneal injection of DMOG 48 hours before a skin ischemic insult could improve skin flap survival [44]. Fewer apoptotic cells were present in the ischemic flaps of DMOG-treated mice. Marked increases in circulating endothelial progenitor cells and bone marrow proliferative progenitor cells were observed after DMOG treatment [44]. We can now consistently show that modulation of prolyl hydroxylases provides a new method of triggering endogenous beneficial mechanisms without applying hypoxic treatments.

Current strategies of BMSC transplantation achieve only modest recovery of cardiac deficits largely because of acute-phase cell death after implantation into the infarcted myocardium [3]. One limitation is that, as multipotent cells, the transdifferentiation efficiency of BMSCs into cardiac lineage cells is low and their engraftment in the ischemic heart is not clear [45]. The present investigation focuses on the survival and increased paracrine factors in preconditioned cells. Whether transplanted BMSCs became cardiomyocytes or vascular endothelial cells and whether DMOG preconditioning enhances the transdifferentiation *in vivo* remain to be examined.

In cell transplantation therapy, besides selections of ideal timing and the route of cell transplantation, it has also become important to test gene modification and cell preconditioning for increased cell survival [2, 7, 25, 46, 47]. A concern with regards to gene modification is whether or not permanent gene modification would increase the risk of tumorigenesis. Permanent or long-term gene modification in cell-based therapy may therefore be of limited use in clinical applications [48]. Since preconditioning strategies usually modify related genes in a relatively short period (days to weeks), this approach may be more feasible for clinical applications. To this end, hypoxic preconditioning has been shown highly effective in transplantation therapy using several stem cells and neural progenitor cells after ischemic stroke and other disorders [21, 39]. For example, our group was among the first to report that hypoxic preconditioning could be applied to stem cell therapy for enhanced cell survival and optimized regenerative capabilities including enhanced angiogenesis, neurogenesis, suppressing inflammatory response, improved directed cell migration, homing to the ischemic region, and finally better functional recovery after heart and brain ischemia [7, 15–17]. Since then a number of preconditioning mediators and pharmacological reagents have been tested for the preconditioning of different cells [18, 19].

Our previous findings showed that both the HIF-1α pathway and the PI3K/Akt signaling were involved in the DMOG protective mechanisms [37]. In accordance with this, we found HIF-1α and Akt pathways were both activated in DMOG-BMSCs. HIF-1α plays an important role in vascular development and embryonic lethality. In HIF-1α^-/-^ mice, defects in angiogenesis have been observed in both the yolk sac and the developing embryonic tissues [49]. HIF-1α stabilization and enhanced VEGF expression followed by prolyl hydroxylase inhibition increased lung angiogenesis in the primate model of bronchopulmonary dysplasia, a chronic form of lung disease [50]. HIF-1α stabilization is a major stimulus for increased VEGF production that plays a pivotal role in angiogenesis. Activation of the PI3K/Akt pathway can also increase VEGF secretion both by HIF-1-dependent and HIF-1-independent mechanisms [51]. These observations are consistent with our observation that HIF-1α and the PI3K/Akt pathway, as well as some downstream molecules such as VEGF, contribute to the protective and regenerative benefits with DMOG preconditioning of BMSCs.

## Conclusion

We conclude that targeting an oxygen sensing system such as prolyl hydroxylase provides a new promising pharmacological approach for enhanced survival of BMSCs, increased paracrine signaling, augmented regenerative activities and improved functional recovery in cell transplantation therapy for the ischemic heart. The present data and previous evidence support a possible preconditioning benefit of increased transdifferentiation of BMSCs into cardiac lineage cells.

## Authors’ information

XL is currently an MD in the Department of Cardiology, Second Affiliated Hospital, Zhejiang University, China. J-AW is the Director of the Cardiovascular Center, Dean of the Second Affiliated Hospital, Zhejiang University, China. X-YJ is a Research Fellow at Emory University, USA. SPY is an O. Wayne Rollins Endowed Chair Professor at Emory University, USA. LW is a John E. Steinhaus Endowed Chair Professor at Emory University, USA.

## Abbreviations

BMSC: bone marrow mesenchymal stem cell
C-BMSCs: control BMSCs
DMOG: dedimethyloxalylglycine
DMOG-BMSCs: DMOG-preconditioned BMSCs
EPO: erythropoietin
Glut-1: glucose transporter 1
HIF: hypoxia-inducible factor
LVEDP: left ventricular end-diastolic pressure
LVEF: left ventricular ejection fraction
LVSP: left ventricular systolic pressure
MI: myocardial infarction
PBS: phosphate-buffered saline solution
PI3K: phosphatidylinositol-4,5-bisphosphate 3-kinase
TUNEL: terminal deoxynucleotidyl transferase biotin-dUPT nick end labeling
VEGF: vascular endothelial growth factor.

## References

[1] Braunwald E, Bristow MR (2000). Congestive heart failure: fifty years of progress. Circulation.

[2] Williams AR, Hare JM (2011). Mesenchymal stem cells: biology, pathophysiology, translational findings, and therapeutic implications for cardiac disease. Circulation Res.

[3] Tang YL, Tang Y, Zhang YC, Qian K, Shen L, Phillips MI (2005). Improved graft mesenchymal stem cell survival in ischemic heart with a hypoxia-regulated heme oxygenase-1 vector. J Am College Cardiol.

[4] Bartunek J, Croissant JD, Wijns W, Gofflot S, de Lavareille A, Vanderheyden M, Kaluzhny Y, Mazouz N, Willemsen P, Penicka M, Mathieu M, Homsy C, De Bruyne B, McEntee K, Lee IW, Heyndrickx GR (2007). Pretreatment of adult bone marrow mesenchymal stem cells with cardiomyogenic growth factors and repair of the chronically infarcted myocardium. Am J Physiol Heart Circ Physiol.

[5] Behfar A, Yamada S, Crespo-Diaz R, Nesbitt JJ, Rowe LA, Perez-Terzic C, Gaussin V, Homsy C, Bartunek J, Terzic A (2010). Guided cardiopoiesis enhances therapeutic benefit of bone marrow human mesenchymal stem cells in chronic myocardial infarction. J Am Coll Cardiol.

[6] McGinley LM, McMahon J, Stocca A, Duffy A, Flynn A, O'Toole D, O'Brien T (2013). Mesenchymal stem cell survival in the infarcted heart is enhanced by lentivirus vector-mediated heat shock protein 27 expression. Human Gene Ther.

[7] Wei L, Fraser JL, Lu ZY, Hu X, Yu SP (2012). Transplantation of hypoxia preconditioned bone marrow mesenchymal stem cells enhances angiogenesis and neurogenesis after cerebral ischemia in rats. Neurobiol Dis.

[8] Wang L, Pasha Z, Wang S, Li N, Feng Y, Lu G, Millard RW, Ashraf M (2013). Protein kinase G1 alpha overexpression increases stem cell survival and cardiac function after myocardial infarction. PLoS One.

[9] Kim SW, Lee DW, Yu LH, Zhang HZ, Kim CE, Kim JM, Park TH, Cha KS, Seo SY, Roh MS, Lee KC, Jung JS, Kim MH (2012). Mesenchymal stem cells overexpressing GCP-2 improve heart function through enhanced angiogenic properties in a myocardial infarction model. Cardiovasc Res.

[10] Hu X, Yu SP, Fraser JL, Lu Z, Ogle ME, Wang JA, Wei L (2008). Transplantation of hypoxia-preconditioned mesenchymal stem cells improves infarcted heart function via enhanced survival of implanted cells and angiogenesis. J Thorac Cardiovasc Surg.

[11] Kim HW, Haider HK, Jiang S, Ashraf M (2009). Ischemic preconditioning augments survival of stem cells via miR-210 expression by targeting caspase-8-associated protein 2. J Biol Chem.

[12] Chacko SM, Ahmed S, Selvendiran K, Kuppusamy ML, Khan M, Kuppusamy P (2010). Hypoxic preconditioning induces the expression of prosurvival and proangiogenic markers in mesenchymal stem cells. Am J Physiol Cell Physiol.

[13] Jaussaud J, Biais M, Calderon J, Chevaleyre J, Duchez P, Ivanovic Z, Couffinhal T, Barandon L (2013). Hypoxia-preconditioned mesenchymal stromal cells improve cardiac function in a swine model of chronic myocardial ischaemia. Eur J Cardiothorac Surg.

[14] Tang YL, Zhu W, Cheng M, Chen L, Zhang J, Sun T, Kishore R, Phillips MI, Losordo DW, Qin G (2009). Hypoxic preconditioning enhances the benefit of cardiac progenitor cell therapy for treatment of myocardial infarction by inducing CXCR4 expression. Circ Res.

[15] Francis KR, Wei L (2011). Human embryonic stem cell neural differentiation and enhanced cell survival promoted by hypoxic preconditioning. Cell Death Dis.

[16] Hu X, Wei L, Taylor TM, Wei J, Zhou X, Wang JA, Yu SP (2011). Hypoxic preconditioning enhances bone marrow mesenchymal stem cell migration via Kv2.1 channel and FAK activation. Am J Physiol Cell Physiol.

[17] Theus MH, Wei L, Cui L, Francis K, Hu X, Keogh C, Yu SP (2008). In vitro hypoxic preconditioning of embryonic stem cells as a strategy of promoting cell survival and functional benefits after transplantation into the ischemic rat brain. Exp Neurol.

[18] Wei N, Yu SP, Gu X, Taylor TM, Song D, Liu XF, Wei L (2012). Delayed intranasal delivery of hypoxic-preconditioned bone marrow mesenchymal stem cells enhanced cell homing and therapeutic benefits after ischemic stroke in mice. Cell Transplant.

[19] Haider H, Ashraf M (2008). Strategies to promote donor cell survival: combining preconditioning approach with stem cell transplantation. J Mol Cell Cardiol.

[20] Kohin S, Stary CM, Howlett RA, Hogan MC (2001). Preconditioning improves function and recovery of single muscle fibers during severe hypoxia and reoxygenation. Am J Physiol Cell Physiol.

[21] Yu SP, Wei Z, Wei L (2013). Preconditioning strategy in stem cell transplantation therapy. Transl Stroke Res.

[22] Haider KH, Ashraf M (2012). Preconditioning approach in stem cell therapy for the treatment of infarcted heart. Prog Mol Biol Transl Sci.

[23] Li SC, Acevedo J, Wang L, Jiang H, Luo J, Pestell RG, Loudon WG, Chang AC (2012). Mechanisms for progenitor cell-mediated repair for ischemic heart injury. Curr Stem Cell Res Ther.

[24] Mottaghi S, Larijani B, Sharifi AM (2012). Apelin 13: a novel approach to enhance efficacy of hypoxic preconditioned mesenchymal stem cells for cell therapy of diabetes. Med Hypotheses.

[25] Sakata H, Niizuma K, Wakai T, Narasimhan P, Maier CM, Chan PH (2012). Neural stem cells genetically modified to overexpress cu/zn-superoxide dismutase enhance amelioration of ischemic stroke in mice. Stroke.

[26] Zeng X, Yu SP, Taylor T, Ogle M, Wei L (2012). Protective effect of apelin on cultured rat bone marrow mesenchymal stem cells against apoptosis. Stem Cell Res.

[27] Afzal MR, Haider H, Idris NM, Jiang S, Ahmed RP, Ashraf M (2009). Preconditioning promotes survival and angiomyogenic potential of mesenchymal stem cells in the infarcted heart via NF-κB signaling. Antioxid Redox Signal.

[28] Niagara MI, Haider H, Jiang S, Ashraf M (2007). Pharmacologically preconditioned skeletal myoblasts are resistant to oxidative stress and promote angiomyogenesis via release of paracrine factors in the infarcted heart. Circ Res.

[29] Nagy K, Kis B, Rajapakse NC, Bari F, Busija DW (2004). Diazoxide preconditioning protects against neuronal cell death by attenuation of oxidative stress upon glutamate stimulation. J Neurosci Res.

[30] Bao W, Qin P, Needle S, Erickson-Miller CL, Duffy KJ, Ariazi JL, Zhao S, Olzinski AR, Behm DJ, Pipes GC, Jucker BM, Hu E, Lepore JJ, Willette RN (2010). Chronic inhibition of hypoxia-inducible factor prolyl 4-hydroxylase improves ventricular performance, remodeling, and vascularity after myocardial infarction in the rat. J Cardiovasc Pharmacol.

[31] Kim JW, Tchernyshyov I, Semenza GL, Dang CV (2006). HIF-1-mediated expression of pyruvate dehydrogenase kinase: a metabolic switch required for cellular adaptation to hypoxia. Cell Metab.

[32] Iyer NV, Kotch LE, Agani F, Leung SW, Laughner E, Wenger RH, Gassmann M, Gearhart JD, Lawler AM, Yu AY, Semenza GL (1998). Cellular and developmental control of O_2_ homeostasis by hypoxia-inducible factor 1 alpha. Genes Dev.

[33] Lomb DJ, Straub JA, Freeman RS (2007). Prolyl hydroxylase inhibitors delay neuronal cell death caused by trophic factor deprivation. J Neurochem.

[34] Ogle ME, Gu X, Espinera AR, Wei L (2011). Inhibition of prolyl hydroxylases by dimethyloxaloylglycine after stroke reduces ischemic brain injury and requires hypoxia inducible factor-1α. Neurobiol Dis.

[35] Rey S, Luo W, Shimoda LA, Semenza GL (2011). Metabolic reprogramming by HIF-1 promotes the survival of bone marrow-derived angiogenic cells in ischemic tissue. Blood.

[36] Sasabe E, Tatemoto Y, Li D, Yamamoto T, Osaki T (2005). Mechanism of HIF-1α -dependent suppression of hypoxia-induced apoptosis in squamous cell carcinoma cells. Cancer Sci.

[37] Liu XB, Wang JA, Ogle ME, Wei L (2009). Prolyl hydroxylase inhibitor dimethyloxalylglycine enhances mesenchymal stem cell survival. J Cell Biochem.

[38] Campagnoli C, Roberts IA, Kumar S, Bennett PR, Bellantuono I, Fisk NM (2001). Identification of mesenchymal stem/progenitor cells in human first-trimester fetal blood, liver, and bone marrow. Blood.

[39] Cai H, Zhang Z, Yang GY (2014). Preconditioned stem cells: a promising strategy for cell-based ischemic stroke therapy. Curr Drug Targets.

[40] Min JY, Sandmann S, Meissner A, Unger T, Simon R (1999). Differential effects of mibefradil, verapamil, and amlodipine on myocardial function and intracellular Ca(2+) handling in rats with chronic myocardial infarction. J Pharmacol Exp Ther.

[41] Misra MK, Sarwat M, Bhakuni P, Tuteja R, Tuteja N (2009). Oxidative stress and ischemic myocardial syndromes. Med Sci Monit.

[42] Leenen FH, Huang BS, Yu H, Yuan B (1995). Brain 'ouabain' mediates sympathetic hyperactivity in congestive heart failure. Circ Res.

[43] Ockaili R, Natarajan R, Salloum F, Fisher BJ, Jones D, Fowler AA, Kukreja RC (2005). HIF-1 activation attenuates postischemic myocardial injury: role for heme oxygenase-1 in modulating microvascular chemokine generation. Am J Physiol Heart Circ Physiol.

[44] Takaku M, Tomita S, Kurobe H, Kihira Y, Morimoto A, Higashida M, Ikeda Y, Ushiyama A, Hashimoto I, Nakanishi H, Tamaki T (2012). Systemic preconditioning by a prolyl hydroxylase inhibitor promotes prevention of skin flap necrosis via HIF-1-induced bone marrow-derived cells. PLoS One.

[45] Psaltis PJ, Zannettino AC, Worthley SG, Gronthos S (2008). Concise review: mesenchymal stromal cells: potential for cardiovascular repair. Stem Cells.

[46] Olson SD, Pollock K, Kambal A, Cary W, Mitchell GM, Tempkin J, Stewart H, McGee J, Bauer G, Kim HS, Tempkin T, Wheelock V, Annett G, Dunbar G, Nolta JA (2012). Genetically engineered mesenchymal stem cells as a proposed therapeutic for Huntington's disease. Mol Neurobiol.

[47] Yu X, Chen D, Zhang Y, Wu X, Huang Z, Zhou H, Zhang Z (2012). Overexpression of CXCR4 in mesenchymal stem cells promotes migration, neuroprotection and angiogenesis in a rat model of stroke. J Neurol Sci.

[48] Meuillet EJ, Mahadevan D, Vankayalapati H, Berggren M, Williams R, Coon A, Kozikowski AP, Powis G (2003). Specific inhibition of the Akt1 pleckstrin homology domain by D-3-deoxy-phosphatidyl-myo-inositol analogues. Mol Cancer Ther.

[49] Kotch LE, Iyer NV, Laughner E, Semenza GL (1999). Defective vascularization of HIF-1alpha-null embryos is not associated with VEGF deficiency but with mesenchymal cell death. Dev Biol.

[50] Asikainen TM, Waleh NS, Schneider BK, Clyman RI, White CW (2006). Enhancement of angiogenic effectors through hypoxia-inducible factor in preterm primate lung in vivo. Am J Physiol Lung Cell Mol Physiol.

[51] Jiang BH, Liu LZ (2009). PI3K/PTEN signaling in angiogenesis and tumorigenesis. Adv Cancer Res.

